# Chloroacetate esterase reaction combined with immunohistochemical characterization of the specific tissue microenvironment of mast cells

**DOI:** 10.1007/s00418-022-02174-1

**Published:** 2023-01-04

**Authors:** Dmitri Atiakshin, Andrey Kostin, Igor Buchwalow, Denis Morozow, Vera Samoilova, Markus Tiemann

**Affiliations:** 1 Research and Educational Resource Center for Immunophenotyping, Digital Spatial Profiling and Ultrastructural Analysis Innovative Technologies, Peoples’ Friendship University of Russia, Moscow, Russia; 2 Research Institute of Experimental Biology and Medicine, Burdenko Voronezh State Medical University, Voronezh, Russia; 3 Institute for Hematopathology, Fangdieckstr. 75a, 22547 Hamburg, Germany

**Keywords:** Mast cells, Chloroacetate esterase, Immunohistochemistry, Enzyme histochemistry, Specific tissue microenvironment

## Abstract

This study provides a combined histochemical method for detecting enzyme activity of chloroacetate esterase simultaneously with immunolabeling of the components of a specific tissue microenvironment on formalin-fixed, paraffin-embedded specimens. Chromogenic detection of the molecular targets within and outside the mast cells provides novel options in determining the histoarchitectonics of organ-specific mast cell populations, studying the functional significance of chloroacetate esterase and specifying the immune landscape of the tissue microenvironment.

## Introduction

The chloroacetate esterase (ChAE) is a specific esterase (no E.C. number assigned) that hydrolyzes chloroacetyl esters of unsubstituted and substituted naphthols. Naphthol-AS-chloroacetate was first used by Gomori as a substrate for cytochemical identification of the cytoplasmic esterase activity (Gomori 1953). Later a more suitable substrate—naphthol AS-D-chloroacetate—was found (Moloney et al. 1960; Rindler et al. 1971). Naphthol AS-D-chloroacetate became the substrate of choice for the ChAE assay in enzyme histochemistry (Leder 1979). As a result of the hydrolysis of naphthol AS-D-chloroacetate, free naphthols subsequently interact with diazonium compounds, forming a brightly colored dye at the sites of the enzyme activity.

A histochemical technique developed by Leder (1979) permitted the detection of ChAE activity in neutrophilic granulocytes and mast cells (MCs). A high activity of ChAE was also found in the bone marrow in promyelocytes, gradually decreasing with differentiation. ChAE activity is preserved in tumor cells of the granulocytic lineage (Chinprasertsuk et al. 1991). The detectable presence of ChAE in other cells, in particular, monocytes, macrophages, lymphocytes, megakaryocytes, is controversial (Wachstein and Wolf 1958; Milicevic and Milicevic 1985). In neutrophilic leukocytes, ChAE is contained in primary granules (Frederix and Baert 1986). The enzyme is found in much greater amounts in MC granules (Wong et al. 1982; Atiakshin et al. 2017). High resistance to formalin fixation, as well as to other fixatives, has led to the widespread use of ChAE as a MC marker in histological and pathomorphological studies (Michova and Fakan 1980; Hahn von Dorsche et al. 1981; Lam et al. 1985; Kashima et al. 2015; Atiakshin et al. 2017; Zushi et al. 2018; Uchihara et al. 2019). The efficiency of MC detection by detecting ChAE activity can be comparable or even exceed other methods for MC detection, including immunohistochemical methods (Atiakshin et al. 2017; Wong et al. 1982). Notably, the sufficient simplicity of identifying the intraorgan MC populations using ChAE does not provide a researcher with sufficient information about the functional parameters of MCs in a particular specific tissue microenvironment. Despite previous studies demonstrating that ChAE has the properties of a chymotrypsin-like enzyme (Benditt and Arase 1959; Li et al. 1973; Rindler et al. 1973), trypsin-like enzyme (Glenner and Cohen 1960), pancreatopeptidase (elastase) (Rindler et al. 1971), cyclooxygenase (Milicevic and Milicevic 1993) and others, the function of the enzyme is still unknown. In addition, there are still no methods to assess the patterns of histotopographic distribution of ChAE-positive mast cells in the immune and stromal landscapes of a specific tissue microenvironment. The present study offers a combination of histochemical methods for assessing the functional significance of ChAE and detailing the immune landscape of the tissue microenvironment in pathological practice and research. ChAE reaction combined with the immunohistochemical characterization of concomitant cells may contribute to the study of specific tissue microenvironment of MC in health and disease.

## Materials and methods

### Case selection

Biomaterial of the skin (*n* = 5), jejunum (*n* = 6) and paraganglioma (*n* = 5) from various patients was taken for diagnostic purposes under informed patient consent. All tissues were immediately formalin-fixed and paraffin-embedded. The paraffin blocks were cut into 2-µm sections, which were subsequently subjected to standard dewaxing and rehydration procedures, following the standard procedure (Buchwalow and Boecker 2010). The samples were retrieved from the files of the Institute for Hematopathology, Hamburg, Germany.

### Ethics

This study was conducted in accordance with the principles of World Medical Association Declaration of Helsinki “Ethical Principles for Medical Research Involving Human Subjects” and approved by the Institutional Review Board of the Institute for Hematopathology, Hamburg, Germany (F-2021-02; 22 January 2021). All subjects gave their informed consent before their inclusion. The samples were redundant clinical specimens that had been de-identified and unlinked from patient information.

### Tissue probe staining

Enzyme activity of ChAE was detected using simultaneous azo-coupling with naphthol AS-D chloroacetate as a substrate and with hexazonium-*p*-rosaniline as a coupling agent according to the standard staining protocol (Leder 1979; Lojda et al. 1979).

After ChAE staining, sections were subjected to antigen retrieval using BOND Epitope Retrieval Solution 1 (Leica Biosystems, Wetzlar, Germany, Cat. No. AR9961). We reported previously that endogenous Fc receptors in routinely fixed cells and tissue probes do not retain their ability to bind Fc fragments of antibodies (Buchwalow et al. 2011); therefore, blocking the endogenous Fc receptors prior to incubation with primary antibodies was omitted.

The list of primary antibodies used in this study is presented in the Table 1. Immunoreaction with primary antibodies was performed using a Ventana Slide Stainer or manually. For manually performed immunostaining, primary antibodies were applied in concentration from 1 to 5 µg/ml (corresponding dilution from 1/500 to 1/5000) and incubated overnight at + 4 °C.

**Table 1:** Primary antibodies used in this study

Antibodies/clone	Host	Source	Concentration
Tryptase #ab2378	Mouse monoclonal Ab	AbCam, United Kingdom	1:5000
Chymase #ab2377	Mouse monoclonal Ab	AbCam, United Kingdom	1:3000
Carboxypeptidase A3 #ab251696	Rabbit monoclonal Ab	AbCam, United Kingdom	1:500
Carboxypeptidase A1 + A2 + B #ab181146	Rabbit monoclonal Ab	AbCam, United Kingdom	1:500
CD8 (SP57)	Rabbit monoclonal Ab	Ventana/Roche, Germany	Ready-to-use
CD20 (L26)	Mouse monoclonal Ab	Ventana/Roche, Germany	Ready-to-use
CD117 (EP10)	Rabbit monoclonal Ab	Leica Biosystems, Germany	Ready-to-use
CD163 (MRQ-26)	Mouse monoclonal Ab	Ventana/Roche, Germany	Ready-to-use
Chromogranin A, (LK2H10)	Mouse monoclonal Ab	Ventana/Roche, Germany	Ready-to-use
S100 (4C4.9)	Mouse monoclonal Ab	Ventana/Roche, Germany	Ready-to-use
Synaptophysin (SP11)	Rabbit monoclonal Ab	Ventana/Roche, Germany	Ready-to-use

Bound primary antibodies were detected with a BOND Polymer Refine Detection Kit (Leica Biosystems, Wetzlar, Germany, Cat. No. DS9800) following visualization using an OptiView DAB IHC Detection Kit (brown color, Cat. No. 760–099) or DISCOVERY Teal HRP (blue color, Cat. No. 760-247), both from Ventana Roche, Mannheim, Germany.

The nuclei were then counterstained with hematoxylin, and the sections were then mounted using Eukitt (Merck, Germany, Cat. No. 03989).

### Controls

Control incubations were omission of primary antibodies or substitution of primary antibodies by the same IgG species (Dianova, Hamburg, Germany) at the same final concentration as the primary antibodies. The exclusion of either the primary or the secondary antibody from the immunohistochemical reaction, substitution of primary antibodies with the corresponding IgG at the same final concentration resulted in lack of immunostaining. Moreover, the specific and selective staining of different cells with the use of primary antibodies from the same species on the same preparation is by itself a sufficient control of the specificity of immunostaining.

### Image acquisition

**Fig. 1:**
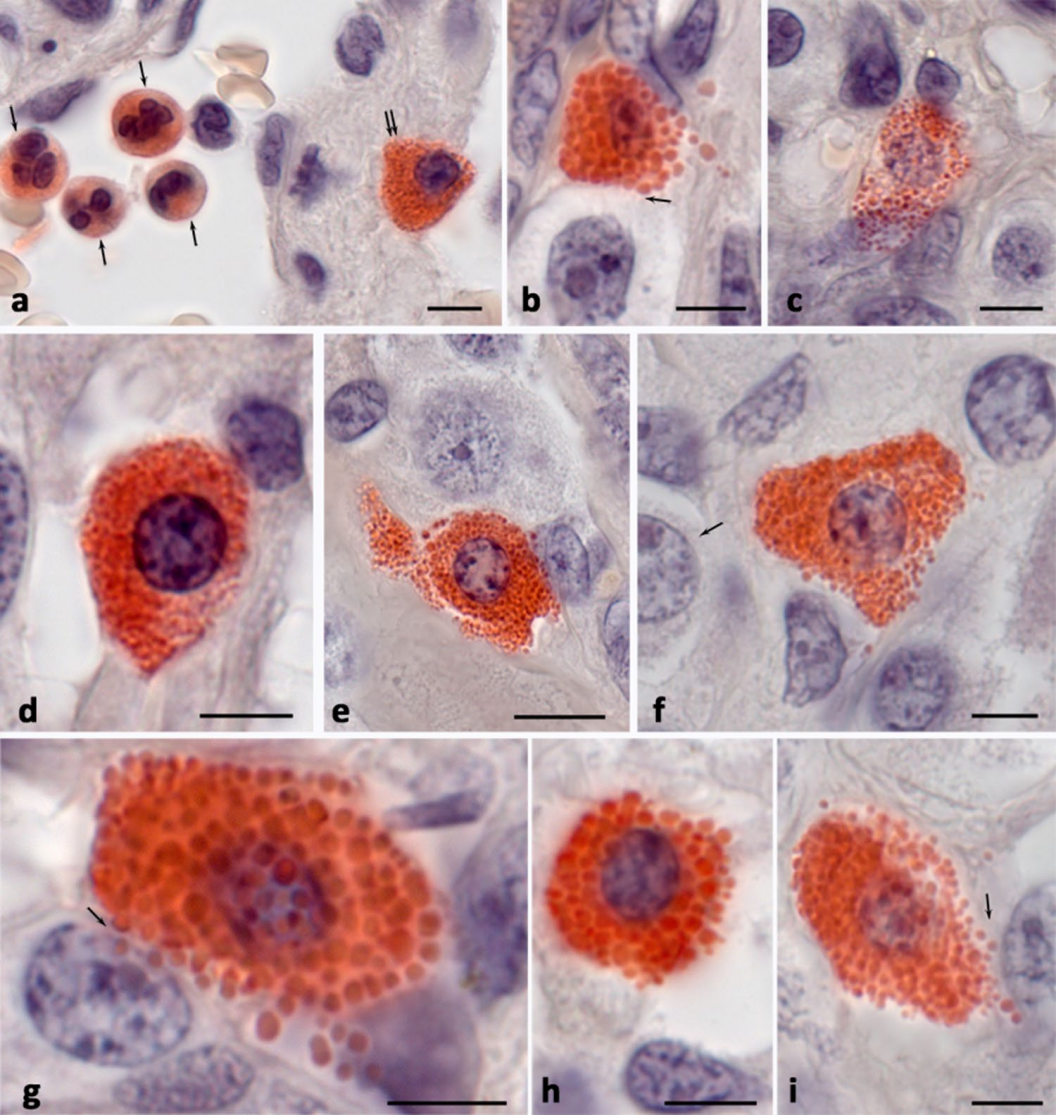
ChAE activity in mast cells. Submucosa of the jejunum (**a**, **c–e**). Paraganglioma (**b**, **f–i**). **a** Positive reaction for ChAE in neutrophil granulocytes (indicated by an arrow) and mast cells (indicated by a double arrow). **b** A MC filled with granules, with signs of secretion of various size granules. Contact of a mast cell with a tumor cell over a large extend (indicated by an arrow). **c** A MC with a loose intracellular distribution of granules. **d** A MC without signs of secretion. **e** Constriction of a part of the MC cytoplasm with the preservation of ChAE activity. **f–i** Proximity of MCs to tumor cells (indicated by an arrow). Scale: 5 µm

**Fig. 2:**
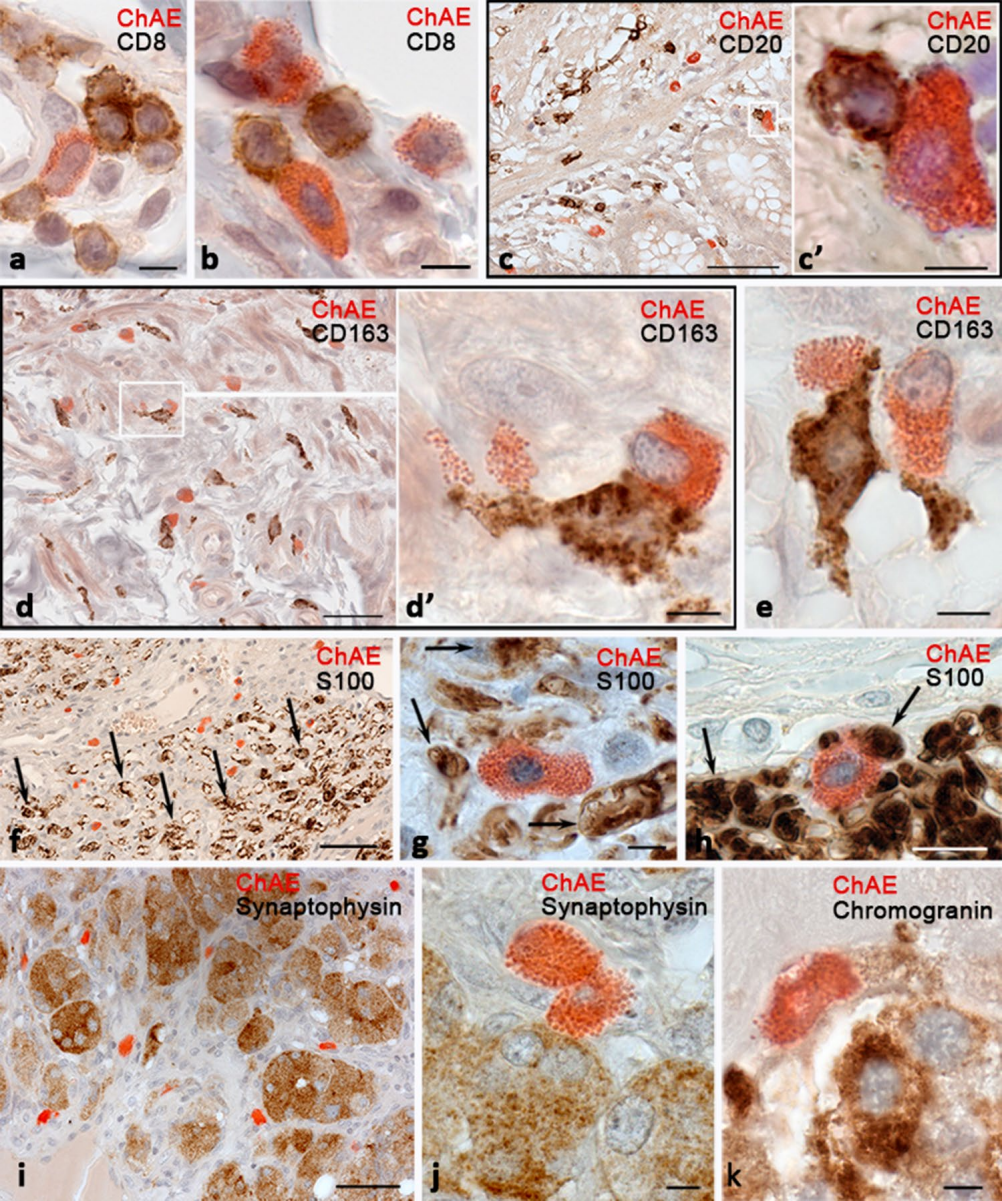
Detection of ChAE activity with immunohistochemical detection of molecular targets absent in mast cells. The skin (**a**), intestines (**b–e**), paraganglioma (**f–k**). **a**, **b** MC contacting with CD8 + lymphocytes. **c** Histotopography of MC and CD20 + lymphocytes. **c’** An enlarged fragment (**c**). **d**, **e** Close relationship between MCs and CD163 + macrophages. **d’** An enlarged fragment (**d**). **f–h** Different variants of protein S100 and MCs co-localization. S100-positive nerve fibers are indicated by an arrow. **i**, **j** Colocalization of MCs and paraganglioma cells with synaptophysin expression. **k** A MC is located next to the chromogranin-positive cell. Scale: **c**, **d**, **f**—50 µm, the rest—5 µm

**Fig. 3:**
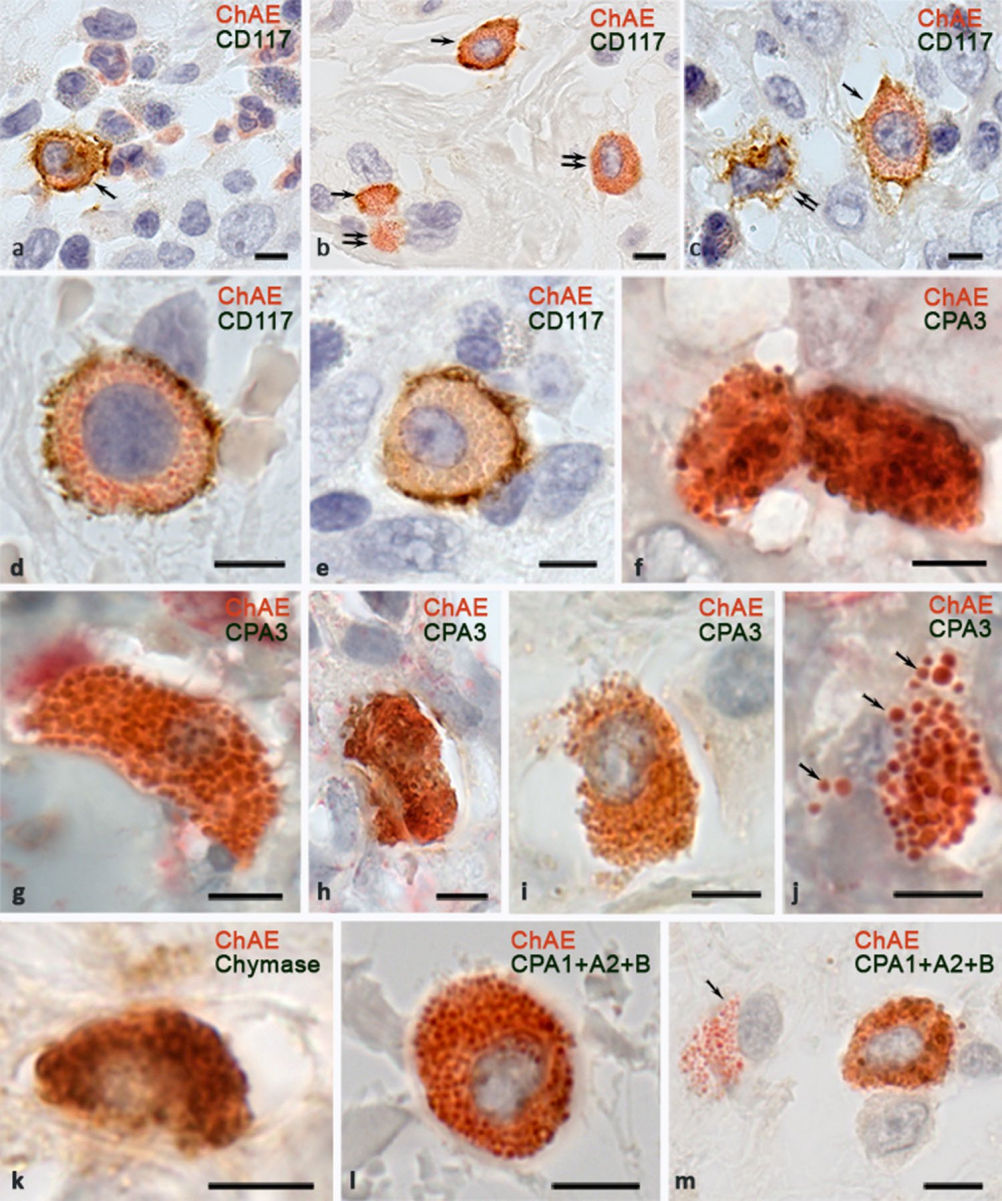
Detection of naphthol AS-D chloroacetate esterase activity with immunohistochemical detection of molecular targets in the mast cell cytoplasm. Intestines (**a–e**, **m**), paraganglioma (**f**, **j**), the skin (**k**, **l**). **a** Only a mast cell is immunopositive for CD117 of all ChAE^+^ cells (arrowed). **b** Different levels of CD117 expression on mast cells and cytoplasmic fragments, from high (indicated by an arrow) to almost complete absence (indicated by a double arrow). **c** A CD117^+^ MC with ChAE activity (indicated by an arrow) and **a** CD117^+^ cell without specific esterase (indicated by double arrow). **d**, **e** A CD117^+^ MC with high (**d**) and low (**e**) levels of ChAE activity. **f** Two MCs contacting with carboxypeptidase A3 expression and ChAE activity. **g** A CPA3^+^ MC filled with large granules, with ChAE activity both within the granules and in the intergranular matrix. In large granules, a peripheral distribution of CPA3 is evident. **h** MCs with ChAE activity and uneven cytoplasmic filling with carboxypeptidase A3 granules. **i** MCs with small secretory granules, some of which contain CPA3. **j** The nuclear-free MC fragment, secretory granules and intergranular matrix have ChAE activity and CPA3 expression. CPA3-positive granules retain ChAE activity after secretion (arrowed). **k** A chymase-positive MC with ChAE activity. **l** A MC containing pancreatic carboxypeptidases has ChAE activity. **m** One of the MCs with ChAE activity does not express CPA1 + A2 + B (indicated by an arrow). Scale: 5 µm

**Fig. 4:**
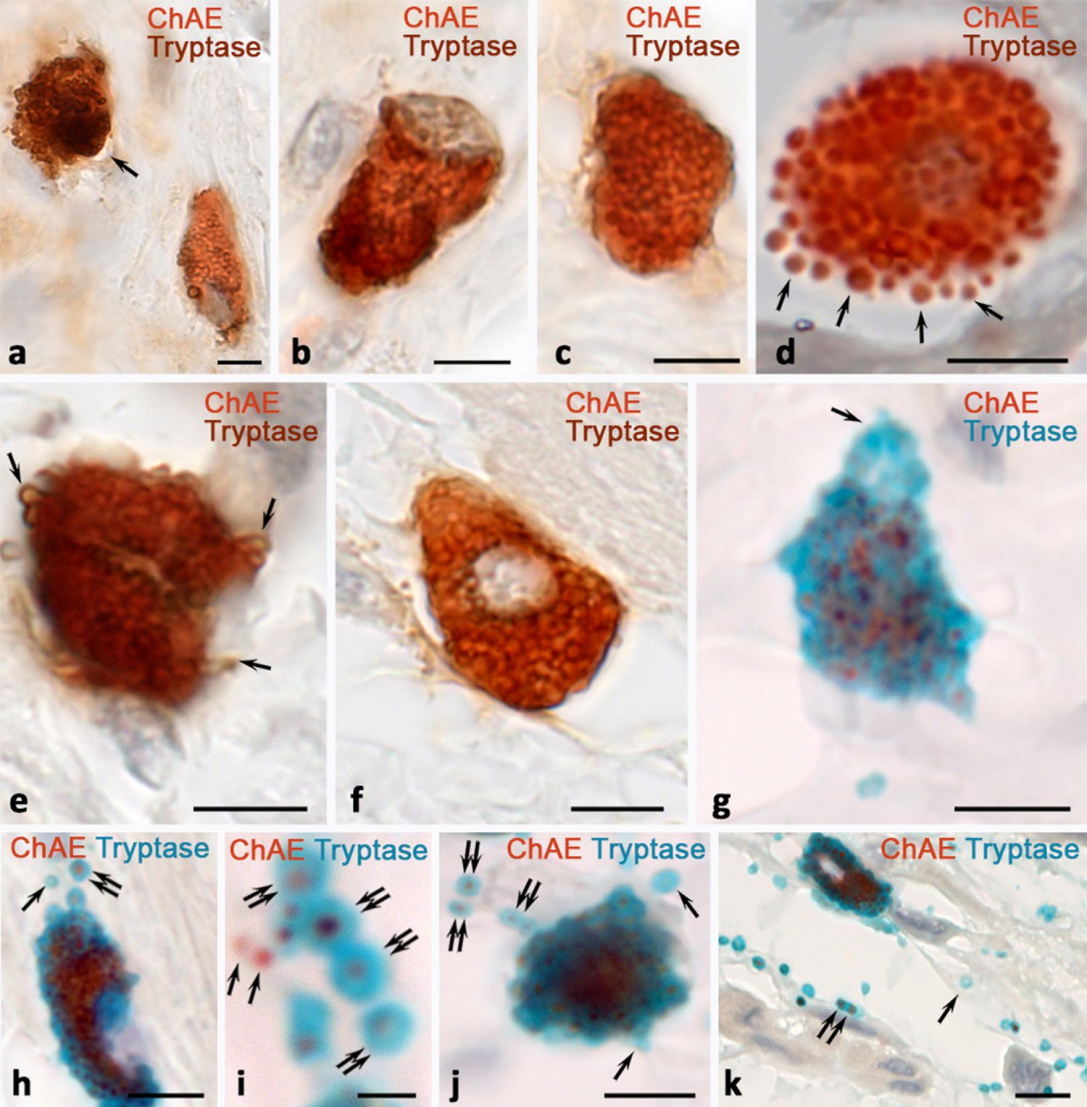
Enzymohistochemical detection of ChAE activity combined with immunohistochemical detection of MC tryptase. Visualization of MC tryptase using an OptiView DAB IHC Detection Kit (brown color) (**a–f**) or DISCOVERY Teal HRP (blue color) (**g–k**). The skin (**a–c**, **h-i**), paraganglioma (**d**, **e**), intestines (**f**, **g**, **j–k**). **a** Two mast cells with ChAE activity, one of which has a higher content of tryptase (indicated by an arrow). **b, c** Mast cells with ChAE activity with focal (**b**) and uniform (**c**) distribution of tryptase in the cytoplasm. **d**, **e** Large MCs with ChAE activity filled with granules and signs of secretory activity (arrowed). In large granules, tryptase localization along the periphery is noticeable. **f** A mast cell with ChAE positive granules and distinct tryptase localization at the periphery of the granules. **g** A tryptase-positive MC, in which ChAE activity is reduced in the region of the cytoplasm that constricts from the cell (indicated by an arrow). **h** A tryptase-positive mast cell with ChAE activity that is retained after secretion in some granules (indicated by double arrow) and is not retained in others (indicated by arrow). **i** Tryptase-negative (arrowed) and tryptase-positive (double-arrowed) MC granules with ChAE activity in the extracellular matrix. **j**, **k** Active degranulation of tryptase-positive mast cells with excretion of secretory granules into the extracellular matrix, some of which lose ChAE activity (indicated by an arrow) and some retain (indicated by a double arrow). Scale − 5 µm

Stained tissue sections were observed on a ZEISS Axio Imager.Z2 equipped with a Zeiss alpha Plan-Apochromat objective 100x/1.46 Oil DIC M27, a Zeiss Objective Plan-Apochromat 150x/1.35 Glyc DIC Corr M27 and a ZEISS Axiocam 712 color digital microscope camera. Captured images were processed with the software programs Zen 3.0 Light Microscopy Software Package, ZEN Module Bundle Intellesis & Analysis for Light Microscopy, ZEN Module Z Stack Hardware (Carl Zeiss Vision, Germany) and submitted with the final revision of the manuscript at 300 DPI (Figs. 1, 2, 3, 4).

## Results

### Detection of ChAE in mast cells

Detection of ChAE on paraffin sections showed intense staining of mast cell granules and intergranular space in the cytoplasm (Fig. 1). Compared to neutrophilic granulocytes, MCs stained more intensely and brightly (Fig. 1a). It was quite easy to distinguish MCs from neutrophilic granulocytes by their larger size, rounded shape of the nucleus, and well-defined granularity of the cytoplasm (Fig. 1a). Notably, MC granules could be large, reaching sizes of 1 µm, or smaller, up to 0.5 µm in size. Granules of various morphometric parameters could be found in the same cell (Fig. 1). As a rule, MCs of the connective tissue type had larger sizes of the cytoplasm and of mature granules (Fig. 1a, b, g–i). In some cases, the MC cytoplasm looked rather pale and fluffy as it was partially filled with ChAE-positive secretory granules (Fig. 1c). Some ChAE-positive MCs were visualized without signs of secretion (Fig. 1d). The degree of staining of the granule area for the enzyme seemed to be uniform, although in large granules one could see a more intense reaction in the peripheral region, apparently associated with the accumulation or enhancement of enzyme activity in this region (Fig. 1b, g). Upon constriction of a cytoplasmic fragment from MC, its structures retained ChAE-positivity. (Fig. 1d).

The granules gradually lost their ChAE activity after secretion (Fig. 1f); it persisted for some time intragranularly and weakened as the granules were secreted from the cell. We noticed that in some cases ChAE activity was high at a considerable distance from the secretion site, which evidenced a significant variability of this trait. The size of secreted ChAE-positive granules could vary significantly from 0.3 μm to 1 μm, which supported dependence of the secretome processing on the state of the tissue microenvironment (Fig. 1b, f–i). Secretion was often accompanied by the MC polarization with the creation of an area for the secretory granules excretion (Fig. 1h).

### Combined staining of ChAE and molecular targets outside the mast cells

The combined approach made it possible to convincingly distinguish MCs from the cells of other immunophenotypes in a specific tissue microenvironment. We tested the potential of detecting MC co-localization with T-killers (CD8^+^) (Fig. 2a, b), B-lymphocytes (CD20^+^) (Fig. 2c), M2 type macrophages (CD163^+^) (Fig. 2d–d', e), and protein S100 (Fig. 2f–h), synaptophysin (Fig. 2i, j), chromogranin (Fig. 2k), protein Ki-67. All combinations of ChAE staining with other molecular markers were able to effectively visualize MC co-localization with subpopulations of other cells, including functional types of T and B lymphocytes, macrophages, neuroendocrine cells, or nerve fibers. Visualization of co-localization was possible both at a low magnification, convenient in pathomorphological practice, and at a high magnification, when appropriate.

### Staining of ChAE and intracellular MC targets

Within a wide range of proteins in MCs, we selected CD117, specific MC proteases (tryptase, chymase, carboxypeptidase A3) (Atiakshin et al. 2018, 2019, 2022), and pancreatic carboxypeptidases; their presence in mast cells was shown earlier (Atiakshin et al. 2022). With simultaneous detection of ChAE and CD117, the brown dye was well visualized on the surface of ChAE MCs (Fig. 3) and is absent in neutrophils (Fig. 3a). Attention was drawn to the presence, albeit in small amounts, of ChAE MCs without CD117 expression (Fig. 3b), and CD117^+^ cells without ChAE (Fig. 3c). The intensity of CD117 expression was different, the fact evidencing morphogenetic lability of MCs, their ability to survive and further differentiate. The intensity of ChAE staining in CD117^+^ MCs was different (Fig. 3d, e), which might be one of the signs of a functional state.

The principal potential of intracellular targets detection following staining with ChAE was shown by specific proteases staining, these proteases being reliable markers of MCs (Figs. 3f–m, 4). It was possible to estimate the number of granules in the cytoplasm of MCs containing specific proteases, and the secretion of each of them into the extracellular matrix. Detection of carboxypeptidase A3 demonstrated predominant intragranular localization in ChAE-positive MCs, regardless of belonging to the connective tissue or mucosal subtype. Concurrently, in large granules, more than 0.7 μm, predominantly peripheral localization of the specific MC protease became noticeable (Fig. 3f, g, j). The secretion of ChAE-positive granules was not always accompanied by the presence of carboxypeptidase A3 (CPA3). Detection of chymase also demonstrated its intracellular location, while in large granules localization of the protease was peripheral (Fig. 3k). Chymase-negative MCs with ChAE activity were also detected. Pancreatic carboxypeptidases were found in some MCs of the studied organs. (Fig. 3i, m). Tryptase was stained in MC granules, both in the cytoplasm and outside during secretion (Fig. 4). The content of tryptase in MCs could be different, which was ultimately reflected in the level of preservation of color features of ChAE chromogenic staining (Fig. 4a). ChAE was detected both in granules and in intergranular areas of the cytoplasm, while tryptase in different parts of the cytoplasm could be stained with the same intensity (Fig. 4b, c).

When using the staining kit DISCOVERY Teal HRP (blue color) (Fig. 4g–k) for visualization of MC tryptase, larger staining areas were detected compared to the OptiView DAB IHC Detection Kit (brown color, Fig. 4a–f) due to a greater diffusion of the dye from the locus of the enzymatic reaction (Fig. 4g–k). ChAE was visualized in the center of the granules, corresponding to the locus of the intragranular location of the serglycin with glycosaminoglycans complex (Fig. 4h–k). With distance from the MCs, after secretion, ChAE activity decrease, and in some granules it was not detected at all (Fig. 4h, j, k) (Fig. 4h, h', j, k, k'). It was possible to see granules without tryptase (Fig. 4i).

## Discussion

Cytochemical staining is an experimental technique based on detection of the expression and distribution of intracellular enzymes or other cell components capable of producing stained products. Combined enzymohistochemical and immunohistochemical staining helps to better understand not only the functional purpose of ChAE, but also the role of MCs in the formation of the immune landscape of a specific tissue microenvironment. Previously, we practiced an integrated approach of metachromatic staining with toluidine blue and immunohistochemical detection of tryptase, which allowed obtaining new data on the structure of the MC population and the features of specific protease biogenesis (Atiakshin et al. 2021). The proposed method for the immunohistochemical detection of molecular targets after the ChAE activity detection has beneficial advantages, first of all, the stability of detection, which allows applying the technique in the analysis of biomaterial of both laboratory animals and humans. In each of the considered organs containing various types of MCs, as well as in the tumor microenvironment, the technique showed similar results, which allows us to conclude that the use of the developed staining design is effective.

As mentioned above, the function of ChAE remains unknown, despite the extensive research in this area. For a long time there has been an opinion about the detoxification and microbiocidal activity of ChAE (Skarnes et al. 1968). ChAE in MCs has been shown to have similar properties to chymotrypsin (Benditt and Arase 1959). Testing the hypothesis of localization of chymotrypsin in MC granules may explain the ChAE functions associated with proteolysis and intracellular degradation of certain substrates. It has been shown that near the wound, MCs are much more likely to lose ChAE activity compared to tryptase content (Oehmichen et al. 2009). This is likely due to the processes of secretion and depletion of certain enzyme systems both in MCs and in granules after their secretion and autonomous stay in a specific tissue microenvironment. Other studies have been related to the study of ChAE in granulocytes. In particular, nine isotypes of esterases were identified in neutrophilic leukocytes; of these, five corresponded to the specific esterase ChAE (Li et al. 1973). Some esterases are most active at various stages of functional differentiation of granulocytes, which allows their features to be used in hematopathology. Interestingly, ChAE-positive neutrophilic granulocytes have a high level of expression of such differentiation clusters as CD11b, CD15 and CD16 (Fedyanina et al. 2016). Obviously, the product of the ChAE detection reaction do not mask intracellular antigens. Currently, techniques allowing detection of not only differentiating eosinophilic, neutrophilic and basophilic alkaline granulocytes during detection of ChAE, but also simultaneously monitoring the activity of ChAE in real time, are being actively developed (Wang et al. 2022).

However, the earlier studies do not provide conclusive evidence for the direct physiological ChAE function. With our approach, it becomes possible to provide immune phenotyping of ChAE-positive MCs in situ with identification of proteins with a known function.

Based on the results of the study performed, it can be assumed that the previously used approach to using the results of ChAE staining to interpret the differentiated content of specific proteases in MCs is biased. MC contain high amounts of tryptase, chymase, and carboxypeptidase A3, as shown by us in previous immunohistochemical studies (Atiakshin et al. 2018, 2019, 2022). We found the presence of both specific proteases (tryptase, chymase, carboxypeptidase A3) and nonspecific pancreatic carboxypeptidases in ChAE-positive MCs. That is why previous studies, in which the reaction to ChAE was associated with particular specific proteases, are at least biased and require revision of the results obtained (Matin et al. 1992). The chymotrypsin-like properties of specific esterases can also be associated with the activity of pancreatic carboxypeptidases, which we found in MCs (Atiakshin et al. 2022). In any case, additional studies are needed to verify specific proteins or their groups, the presence of which can be explained by a positive reaction to ChAE. The proposed method allows targeted identification of enzymes involved in the hydrolysis of naphthol AS-D-chloroacetate. Simultaneous detection of ChAE and intracellular distribution of molecular targets also enables interpreting the selective excretion of MC secretory products.

Interpreting the biology of MCs, it is very important to determine the histotopographic distribution related to other cells and extracellular structures. Concomitantly, this technique is very useful, since it allows us to assess MC integration into the immune or stromal landscape of a specific tissue microenvironment.

Thus, the proposed combined histochemical method for the naphthol AS-D-chloroacetate esterase activity detection significantly expands the systematic potential of specialists investigating MCs in health and disease in the study of biological features of the MC population, the immune landscape of a specific tissue microenvironment, and, finally, in understanding the physiological significance of ChAE.

## Data Availability

All data and materials are available on reasonable request. Address to I.B. (e-mail: buchwalow@pathologie-hh.de) or M.T. (email: mtiemann@hp-hamburg.de) Institute for Hematopathology, Hamburg, Germany.
